# Characteristics of Publications on Occupational Stress: Contributions and Trends

**DOI:** 10.3389/fpubh.2021.664013

**Published:** 2021-06-15

**Authors:** Yang Zhang, Lei Huang, Yongwei Wang, Yajia Lan, Yonggang Zhang

**Affiliations:** ^1^Department of Periodical Press and National Clinical Research Center for Geriatrics, West China Hospital, Sichuan University, Chengdu, China; ^2^Chinese Evidence-Based Medicine Center, West China Hospital, Sichuan University, Chengdu, China; ^3^Department of Environmental Health and Occupational Medicine, West China School of Public Health and West China Fourth Hospital, Sichuan University, Chengdu, China; ^4^Department of Occupational Hazard Assessment, West China School of Public Health and West China Fourth Hospital, Sichuan University, Chengdu, China

**Keywords:** occupational stress, work related, bibliometric analysis, top-cited, citation

## Abstract

This study aimed to analyze the bibliometric characteristics of the publications on occupational stress and highlight key research topics and future trends. The Web of Science Core Collection database was searched to collect publications on occupational stress, from inception to December 9, 2020. Two authors independently screened eligible literature and extracted the data. Bibliometric analyses were performed using VOSviewer 1.6.6 and R 3.6.3 software. Overall, 6,564 publications on occupational stress were included. “Stress,” with a total link strength of 1,252, appeared as the most co-occurrence keyword, followed by “occupational stress,” “job stress,” and “job satisfaction.” All studies were published between 1956 and 2020. Among them, 6,176 (94.35%) papers were written in English, and 4,706 (70.25%) were original articles. The top three Web of Science categories were “public environmental occupational health” (*n* = 1,711), “psychology, applied” (*n* = 846), and “psychology, multidisciplinary” (*n* = 650). The 100 top-cited articles were mentioned a total of 36,145 times, with a median of 361, ranging from 174 to 5,574. The United States was the most productive country, with 1,780 publications. The main partners of the United States were England and China. Three themes of occupational stress research were identified: job satisfaction, burnout, and occupational stress-related health problems. This bibliometric analysis provides a comprehensive understanding of the trends and most influential contributions to the field of occupational stress, thus promoting ideas for future research.

## Introduction

Occupational stress is the process by which workplace psychological experiences and demands (stressors) produce both short- and long-term changes (strains) in mental and physical health (1). The annual National Health Service (NHS) staff survey conducted in England showed that approximately 30% of workers suffer from occupational stress (2). Moreover, a meta-analysis found that the overall prevalence of occupational stress among nurses working in Iranian hospitals was 60% (3). Occupational stress is associated with many diseases (1), such as cardiovascular diseases (4, 5), depression (6), and type 2 diabetes (7). Occupational stress is also closely related to insomnia (8), alcoholism (9), smoking (10), and unhealthy weight fluctuations (11), which further affect physical and mental health.

Recently, increasing research has focused on occupational stress. Bibliometric analysis can detect critical junctures in the development of research fields, especially intellectual turning points and pivotal moments (12). Moreover, it can provide insights regarding prominent areas of interest, with an emphasis on the countries, institutions, journals, and authors with the greatest global impact. The scientific knowledge graph is a new domain in the development of bibliometric analysis, which can visually represent research results (13, 14). Another crucial component of bibliometric analysis is citation analysis, which provides comprehensive information regarding cited articles (15). Bibliometric analysis has been applied in various disciplines, such as neuropsychology (16), cancer (17), respiratory medicine (18), COVID-19 (19), and vaccines (20).

However, no bibliometric analysis of publications has been conducted on occupational stress. Since occupational stress has become an important area of occupational health, and increasing research has been published on the topic, it is imperative to conduct a bibliometric analysis in this area. Therefore, in the present study, we aimed to provide a broad understanding of occupational stress and to highlight the key research topics, thus promoting ideas for future studies.

## Materials and Methods

### Search Strategy

The Web of Science Core Collection database was searched for publications on occupational stress from inception to December 9, 2020. The search strategies were as follows: [occupational OR job OR job-related OR (job related) OR work-related OR (work related) OR workplace OR (work place) OR professional] AND (stress^*^ OR strain^*^) in the title. The language and literature types were not limited during the process of retrieval. Literatures were excluded on several criteria. First, the title of these literatures had the above-mentioned keywords; however, the content of the literature was about heat stress, muscle strain, oxidative stress, stress fracture, postural stresses, non-work-related posttraumatic stress, work-related physical stress, occupational ergonomics, and mechanical stress in the workplace or occupational groups. In this occupational stress literature bibliometric analysis, environmental stressors refer to psychosocial stressors. Second, literatures were excluded when psychological stress was not work related or occurring in nonworking people, such as students or full-time mothers. Finally, items were excluded when strain referred to a microorganism in the workplace. Two authors independently screened titles, abstracts, and full texts for eligible literature. Disagreements were resolved through discussion or by a third reviewer.

### Data Extraction

Information including the title, publication year, language, author, journal name, affiliation, keywords, document type, Web of Science category, abstract, and total number of citations were identified and recorded by two authors. The results were compared, and disagreements were resolved through discussion or by a third reviewer.

### Statistical Analysis

We used VOSviewer software (version 1.6.6) to analyze the co-authorship, co-occurrence, co-citation, citation, themes, and topics. We also used R software (version 3.6.3) to perform correlation analysis, which was used to evaluate the relationships between the different terms. A *p*-value of ≤ 0.05 (two-tailed) was considered statistically significant.

## Results

### Basic Characteristics of Included Literatures

**Figure 1:**
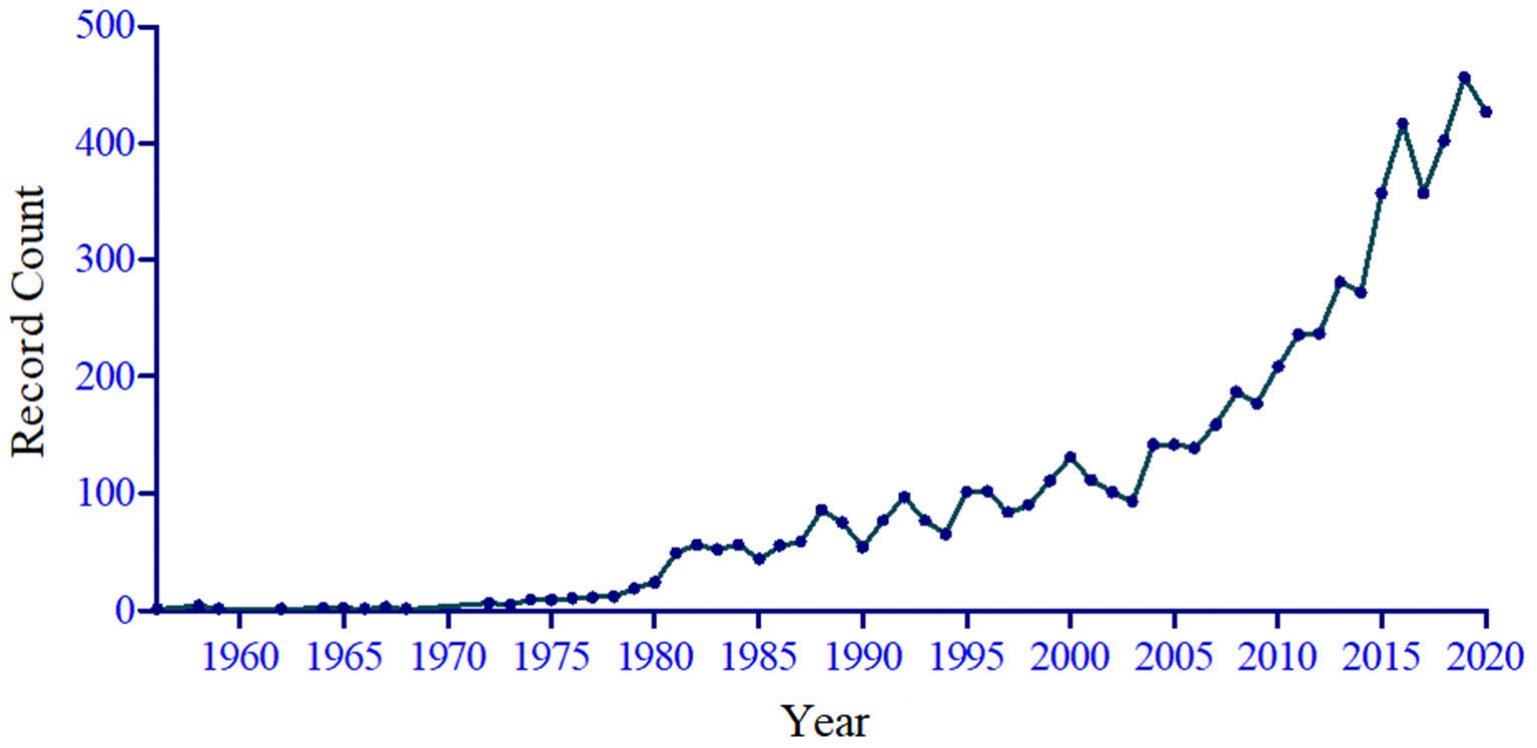
Number of publications issued per year in the occupational stress field from 1956 to 2020.

We identified 7,106 potentially relevant literatures. After screening the titles, abstracts, and full texts, 6,546 publications on the topic of occupational stress were included. The literatures were published between 1956 and 2020 (Figure 1). Among them, 6,176 (94.35%) studies were written in English, followed by German (145; 2.22%) and Spanish (50; 0.76%). Of the published papers, 4,706 (70.25%) were original articles, followed by meeting abstracts (11.29%), proceedings papers (6.84%), reviews (2.81%), book reviews (2.60%), and other forms of publications (6.22%) including editorial material, letters, notes, and so forth. The included publications were divided into different Web of Science categories. The top three Web of Science categories were: “public environmental occupational health” (*n* = 1 711); “psychology, applied” (*n* = 846); and “psychology, multidisciplinary” (*n* = 650).

**Figure 2:**
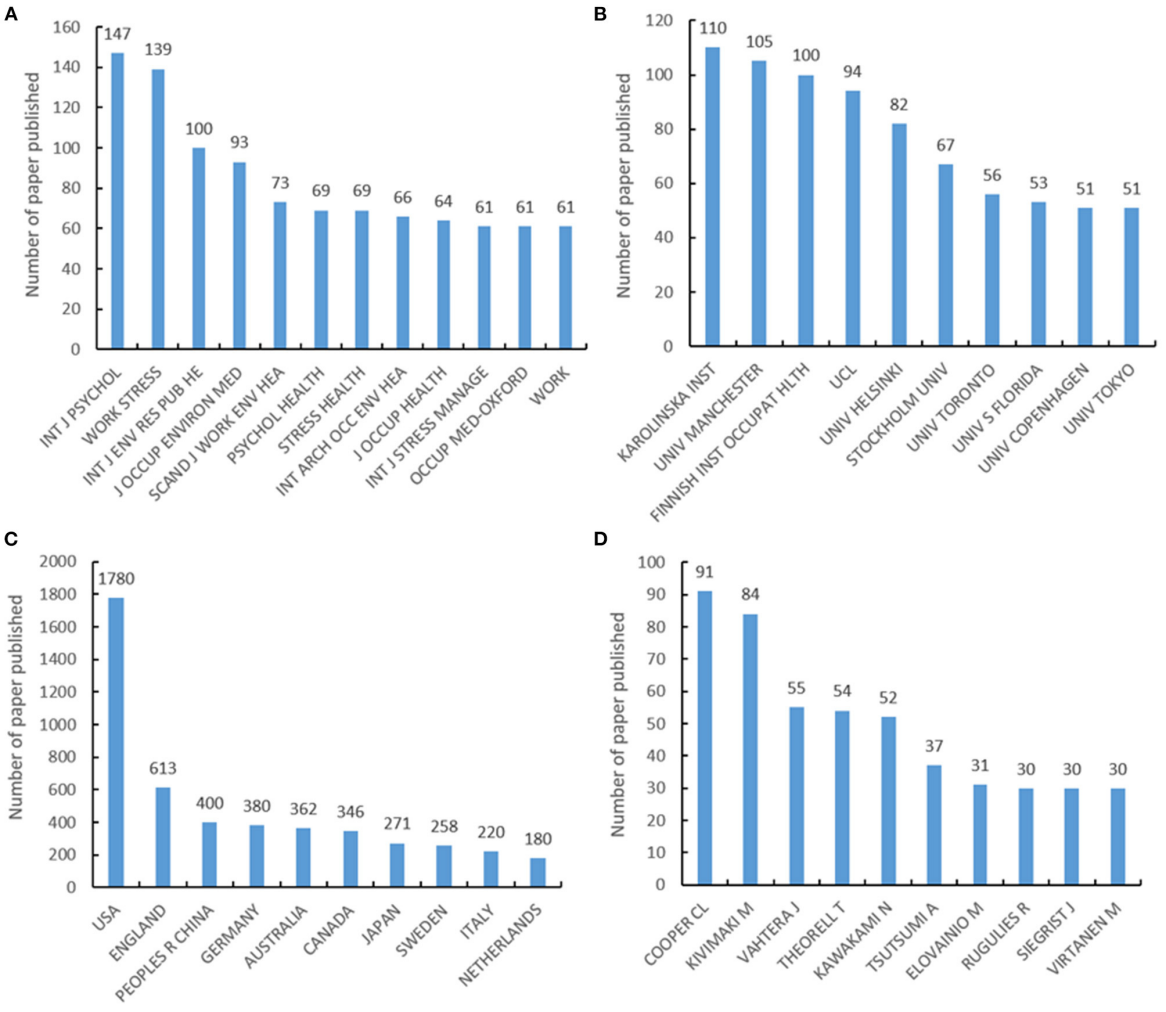
The top 10 most published journals, organizations, countries, and authors of occupational stress publications. **(A)** The top 10 most published journals of occupational stress publications. **(B)** The top 10 most published organizations of occupational stress publications. **(C)** The top 10 most published countries of occupational stress publications. **(D)** The top 10 most published authors of occupational stress publications.

The top 10 most published journals, countries, organizations, and authors for occupational stress are shown in Figure 2. In total, 1,003 (14.82%) papers were published in the top 10 active journals. The *International Journal of Psychology* had the most publications, with an impact factor of 1.255 in 2019. *Work and Stress* ranked second, with an impact factor of 3.512, and ranked in Q1 for the psychology applied category in 2019. A total of 5,618 institutions from 125 countries contributed to the publications on occupational stress. The most productive author was Cooper CL from the University of Manchester, with 91 related papers.

### Bibliometric Analysis of Keywords

**Figure 3:**
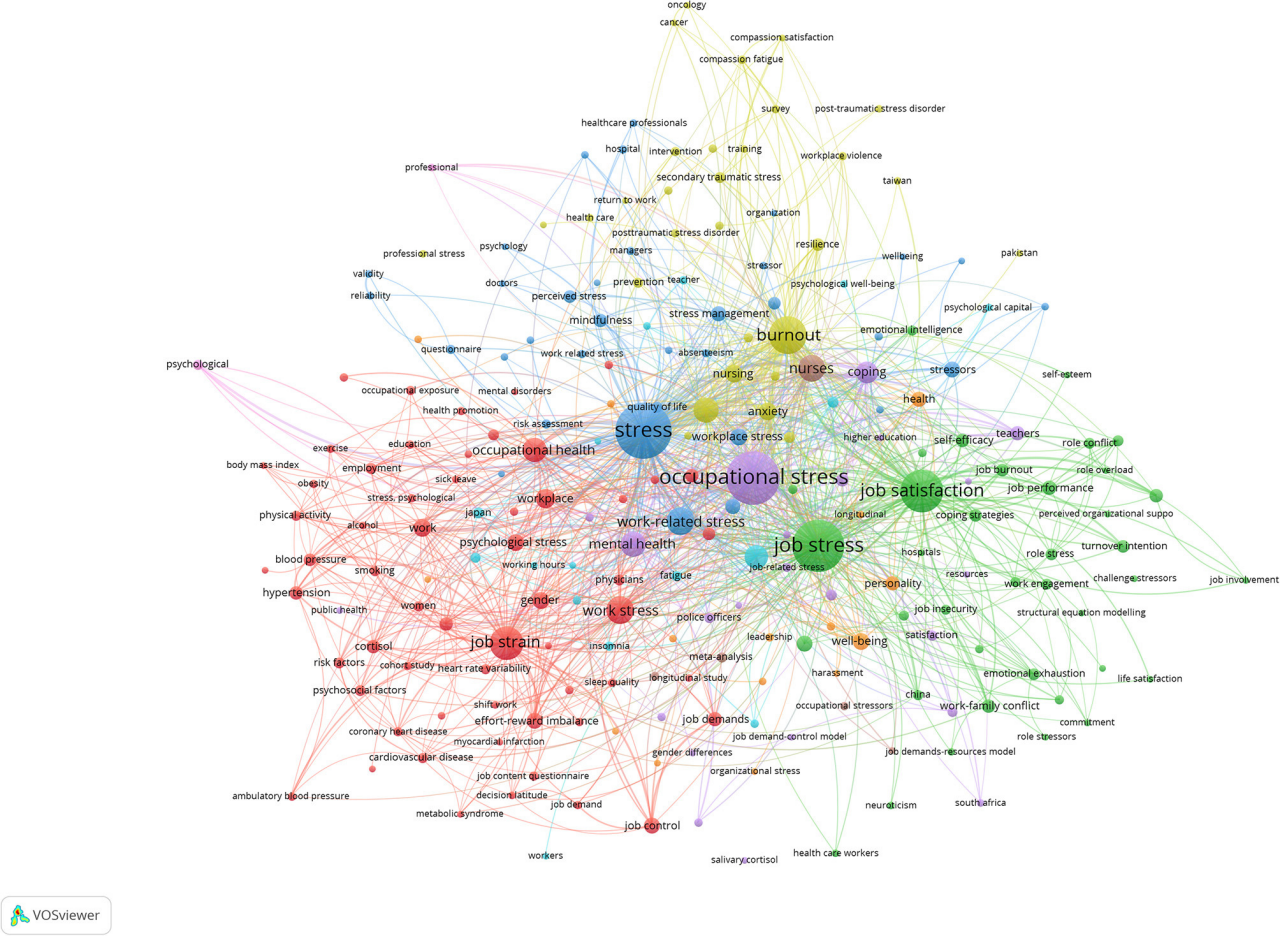
Bibliometric analysis of co-occurrence of keywords in publications of occupational stress. The size of nodes indicates the frequency of occurrence. The curves between the nodes represents their co-occurrence in the same publication. The shorter the distance between two nodes, the larger the number of co-occurrence of the two keywords.

Keywords occurring over 10 times in the database were included in the analysis. Of the 6,818 keywords, 222 met the threshold. “Stress” (with a total link strength of 1 252) appeared as the most co-occurring keyword, followed by “occupational stress,” “job stress,” and “job satisfaction” (Figure 3).

### Bibliometric Analysis of the 100 Top-Cited Papers

The Supplementary Material shows the 100 top-cited papers in the field of occupational stress. The 100 top-cited papers were cited 36,145 times in total, ranging from 174 to 5,574, with a median of 361. The average number of citations per year ranged from 3 to 136, with a median of 18 mentions for the 100 top-cited papers. The total number of citations of the 100 top-cited papers were correlated with the annual mean citations (r = 0.791, p < 0.001). Of the 100 top-cited publications, 93 were original articles, and seven were reviews (Table 1). There were 13 countries that contributed to the 100 top-cited studies. The United States was the most productive country, with the highest average citation. The 100 top-cited studies were published between 1958 and 2015. The most top-cited articles were published in the year 2000 (n = 8), followed by 1998 (n = 7) and 2001 (n = 7). The Journal of Applied Psychology had the highest number of publications (n = 11), followed by the Journal of Organizational Behavior (n = 8), the Scandinavian Journal of Work Environment & Health (n = 5), and the Academy of Management Journal (n = 5).

**Table 1:** Analysis characteristics of the 100 top-cited papers.

**Characteristics**	**Number of studies**	**Total citation**	**Average citation**
**Type of study**			
Article	93	33,669	362
Review	7	2,476	354
**Country**			
USA	55	22,798	415
England	12	4,058	338
Canada	6	1,798	300
Netherlands	5	1,688	338
Germany	5	1,228	246
Sweden	4	1,243	311
Australia	4	1,063	266
Israel	2	718	359
Japan	2	406	203
Greece	2	549	275
Jordan	1	200	200
France	1	199	199
Finland	1	197	197
**Publication year**			
1958	2	647	324
1974	1	198	198
1976	1	578	578
1978	1	520	520
1979	2	5,790	2,895
1980	1	626	626
1981	2	389	195
1982	1	239	239
1983	2	526	263
1984	2	374	187
1986	2	719	360
1987	1	263	263
1988	6	2,938	490
1989	3	901	300
1990	1	344	344
1991	1	177	177
1992	2	386	193
1993	1	268	268
1994	3	1,052	351
1995	2	404	202
1996	2	578	289
1997	2	419	210
1998	7	1,852	265
1999	4	1,037	259
2000	8	2,049	256
2001	7	2,487	355
2003	3	1,052	351
2004	6	1,500	250
2005	3	940	313
2006	1	197	197
2007	4	1,619	405
2008	4	1,591	398
2009	2	523	262
2010	3	1,113	371
2011	3	600	200
2012	3	959	320
2015	1	290	290
Journal^*^			
Journal of applied psychology	11	3,993	363
Journal of organizational behavior	8	2,208	276
Scandinavian journal of work environment & health	5	1,652	330
Academy of management journal	5	1,241	248
American journal of public health	4	2,477	619
Journal of health and social behavior	4	1,236	309
Journal of vocational behavior	4	1,133	283
Journal of occupational health psychology	3	949	316
Work and stress	3	680	227
Personnel psychology	2	985	493
Journal of educational psychology	2	786	393
Lancet	2	716	358
Social science & medicine	2	588	294
Journal of occupational and organizational psychology	2	520	260
Organizational behavior and human performance	2	459	230
Journal of marketing	2	439	220
Psychosomatic medicine	2	437	219

* *Journals that published at least two of the 100 top-cited studies*.

### Bibliometric Analysis of Co-authorship

**Figure 4:**
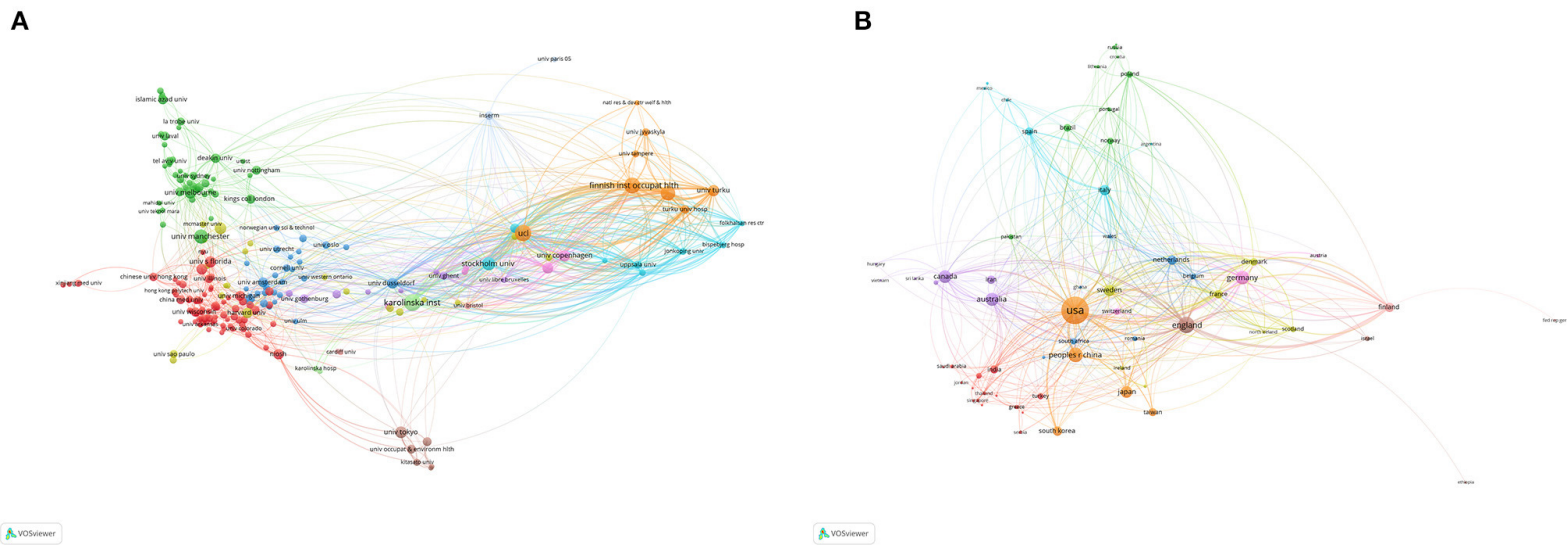
Bibliometric analysis of co-authorship. **(A)** Co-authorship map of organizations. **(B)** Co-authorship map of countries. Different colors indicate different clusters, and the size of circles indicate the number of publications. The thickness of the lines represents the link strength of the organizations or countries.

The co-authorship maps of organizations and countries are shown in Figure 4. The *Karolinska Institutet* published 110 papers. A major partner of the organization was the Stockholm University. The most productive country was the United States with 1,780 publications. Major partners of the United States were England and China.

### Bibliometric Analysis of Co-citation

**Figure 5:**
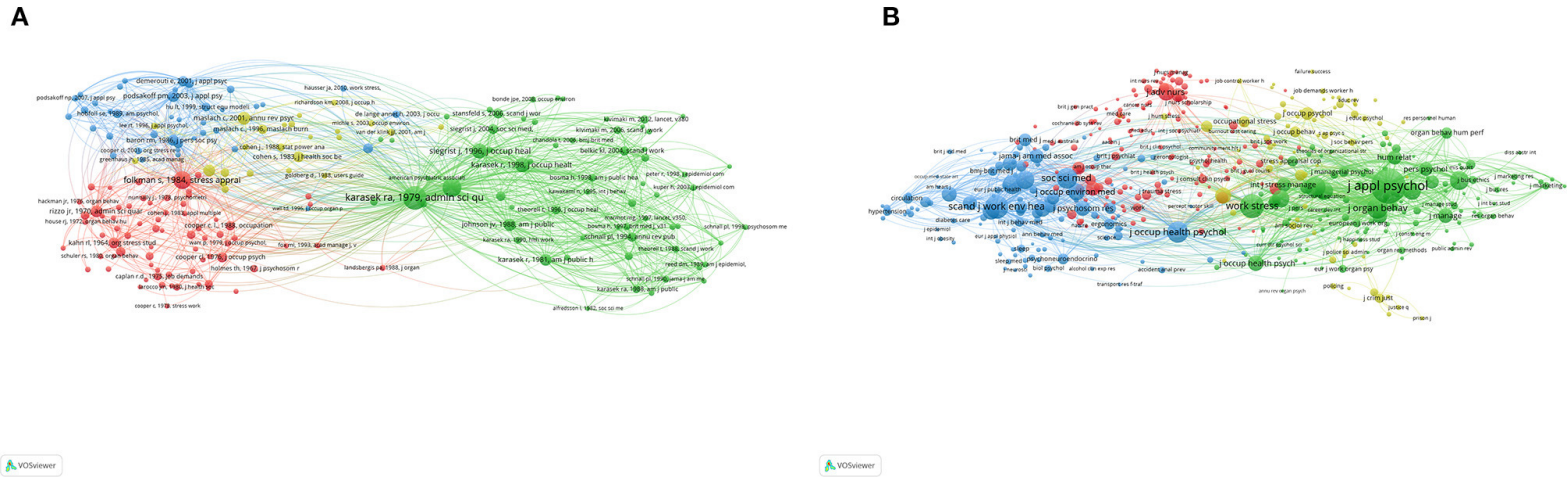
Bibliometric analysis of co-citation. **(A)** Co-citation of the documents. **(B)** Co-citation of the sources. The size of the circles represents the counts of co-citations. The distance between the two circles indicates their correlation.

References with over 40 citations in the database were included in the analysis. Of the 121,510 cited references, 197 met the threshold. Four clusters of the cited references were obtained *via* bibliometric analysis. The most co-cited reference was Karasek RA (1979), with a total link strength of 5,015 and 875 citations (Figure 5A). Sources with over 60 citations in the database were also enrolled into analysis. Of the 38,928 sources, 459 met the threshold. Four clusters of the cited sources were obtained *via* bibliometric analysis. The *Journal of Applied Psychology, Work and Stress*, and the *Journal of Organizational Behavior* ranked in the top three, with 5,760, 2,749, and 2,159 citations, respectively (Figure 5B).

### Bibliometric Analysis of Themes and Trends

**Figure 6:**
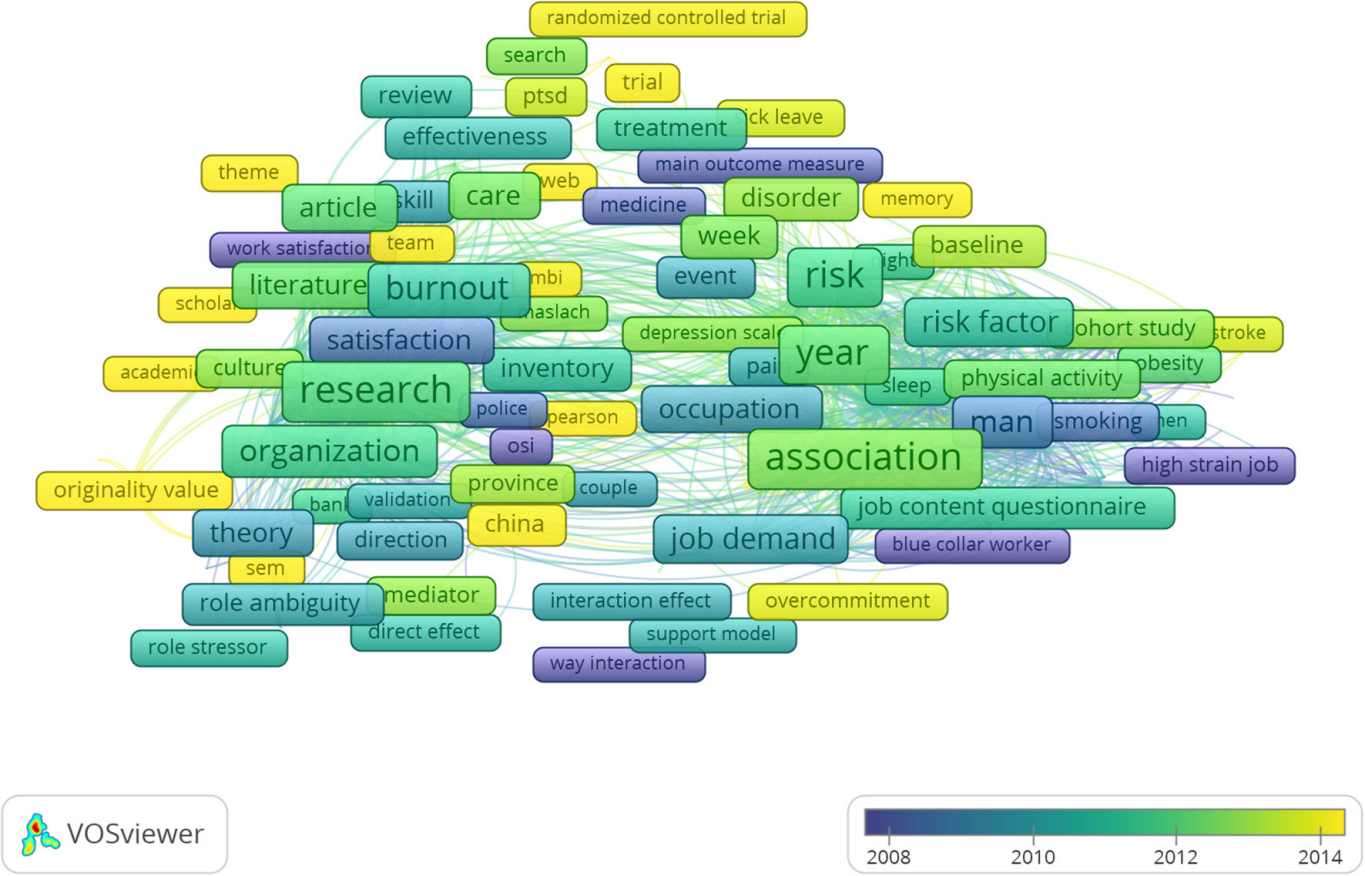
A term co-occurrence map according to terms from the title and abstract fields. Indicators show the current publications from purple to yellow. The size of the frames represents the frequency of appearance as the keywords. The distance between the two frames indicates their correlation. The closer the distance, the more similar the theme.

Terms from the title and abstract fields were extracted. Three themes of occupational stress research were identified: job satisfaction, burnout, and occupational stress-related health problems. Indicators display the current publications from purple to yellow. More researches focusing on stress-related health problems and stress intervention have been published recently (Figure 6).

## Discussion

Bibliometric analysis has been firmly established as a scientific specialty and is an integral component of research evaluation methodology, especially within the scientific and applied fields (21). With the proliferation of medical information, bibliometric analysis has been used to examine various health research domains (22). However, no bibliometric analyses have been conducted among publications on occupational stress. Therefore, this study aimed to provide a comprehensive analysis of published literature in the field of occupational stress.

In this study, we found that the number of literatures published on occupational stress have increased between 1956 and 2020. Occupational stress drastically increased during COVID-19 pandemic, requiring comfort rules for healthcare workers after use of protective device to improve work performance reduction, difficulty in concentrating, and sleep disorders (23). People have begun to emphasize mental health and have realized that occupational stress adversely affects both work performance and overall health (1, 24). Therefore, researchers have increasingly focused on occupational stress. “Stress” appeared as the most co-occurring keyword, followed by “occupational stress,” “job stress,” and “job satisfaction.” Studies concentrated on the following aspects: the theoretical model of occupational stress (1), the stress levels of different occupational groups (25, 26), the relationship between occupational stress and job satisfaction (27), the relationship between occupational stress and disease (1), biomarkers of occupational stress (28), and the relationship between occupational stress and behavioral changes (9). Occupational stress is an interdisciplinary science of public health and psychology. Thus, the top three Web of Science categories were: “public environmental occupational health,” “psychology, applied,” and “psychology, multidisciplinary.”

The most frequently cited article received 5,574 citations and was published in 1979 (29). The major findings of the study indicated that redesigning work processes to allow a greater decision latitude for a broad range of workers could reduce mental strain. This can be achieved without affecting the job demands associated with organizational output levels. The results of the study were widely utilized in subsequent research. Combined with the passage of time, the study had the highest frequency of citations. Meanwhile, the study (29) appeared as the most co-cited reference.

The 100 top-cited studies were not recent, having been published between 1958 and 2015. Similar findings were reported in previous studies (16, 20). It is difficult to obtain high total citations for newly published articles due to the short duration of their availability. Among the 100 top-cited articles, the *Journal of Applied Psychology* had the highest number of publications, followed by the *Journal of Organizational Behavior*. These are two classic journals in the applied psychology field, ranking in the top 10%. While studies on occupational stress have been conducted worldwide, developed countries perform better than developing countries in this research area. The United States was the most productive country with highest average citation. The pronounced influence of the United States may be attributed to its large number of scientific research institutions and abundant research funds. They pay attention to occupational stress earlier and mental health. Major partners of the United States were England and China. Although China ranked third among the most-productive countries, it did not appear among the 100 top-cited articles. This phenomenon indicates that developing countries may disregard occupational stress in favor of devoting more energy to solving physical health problems.

Three themes of occupational stress research were identified: job satisfaction, burnout, and occupational stress-related health problems. Presently, most studies have a cross-sectional design, and few longitudinal studies or pilot trials have been conducted to evaluate health effects and interventions (30, 31). In addition, occupational stress measurement tools are diverse, and the results are difficult to compare. Therefore, more high-quality studies with improved designs focusing on the measurement of occupational stress are needed in the future. More research focusing on stress-related health problems and stress intervention has been published recently. The long-term health effects of occupational stress require more well-designed cohort studies to verify. Furthermore, field intervention trials are needed to evaluate the effects of different occupational stress interventions.

There are some limitations to this study. First, we may have missed literatures that did not contain the keywords in the title. Second, only the Web of Science database was searched for analyses, and we did not collect data from other databases such as Embase, Medline, Scopus, and Google Scholar. Third, the number of citations of each publication will change over time; subsequently, the 100 top-cited articles will also change. Thus, our research is only representative of the current research context. In future studies, we will include more databases in the analyses and dynamically track the changes in this field. Despite these limitations, we believe that as the first bibliometric analysis of occupational stress, our findings make a significant contribution to understanding the trends and influential publications in this domain.

In conclusion, this study analyzed the publications on occupational stress according to the authors, journal, publication year, country, institution, and study type. Moreover, this study provides insight into the 100 top-cited publications in occupational stress research. This bibliometric analysis offers a more comprehensive understanding of trends and the most influential contributions to the field of occupational stress, thus promoting ideas for future research.

## Data Availability Statement

The original contributions presented in the study are included in the article/Supplementary Material, further inquiries can be directed to the corresponding authors.

## Author Contributions

YoZ and YaZ designed the study. YaZ and LH analyzed the data. YaZ drafted the manuscript. LH, YW, YL, and YoZ edited the manuscript. All authors contributed to the article and approved the submitted version.

## Conflict of Interest

The authors declare that the research was conducted in the absence of any commercial or financial relationships that could be construed as a potential conflict of interest.
