# Supplementary material for: Characteristics of Publications on Occupational Stress: Contributions and Trends
Source: Front Public Health. 2021 Jun 15;9:664013. doi: 10.3389/fpubh.2021.664013 (PMC8239141; doi:10.3389/fpubh.2021.664013)
Supplement: Supplementary file 1 [file Table_1.DOCX]

**Supplementary Table 1.** The 100 top-cited studies in occupational stress

| Ranking | Title | Journal | Article type | Total citation | Citation per year | Publication year | country |
| --- | --- | --- | --- | --- | --- | --- | --- |
| 1 | Job demands, job decision latitude, and mental strain - Implications for job redesign | Administrative Science Quarterly | Article | 5574 | 136 | 1979 | USA |
| 2 | Job strain, work place social support, and cardiovascular disease - A cross-sectional study of a random sample of the Swedish working population | American Journal of Public Health | Article | 1265 | 40 | 1988 | USA |
| 3 | Differential challenge stressor-hindrance stressor relationships with job attitudes, turnover intentions, turnover, and withdrawal behavior: A meta-analysis | Journal of Applied Psychology | Article | 763 | 59 | 2007 | USA |
| 4 | Social support, occupational stress, and health | Journal of Health and Social Behavior | Article | 626 | 16 | 1980 | USA |
| 5 | Occupational sources of stress - Review of literature relating to coronary heart-disease and mental ill health | Journal of Occupational Psychology | Review | 578 | 13 | 1976 | England |
| 6 | Job strain and cardiovascular-disease | Annual Review of Public Health | Article | 563 | 22 | 1994 | USA |
| 7 | Effects on teachers' self-efficacy and job satisfaction: Teacher gender, years of experience, and job stress | Journal of Educational Psychology | Article | 560 | 56 | 2010 | Canada |
| 8 | Effects of occupational stress management intervention programs: A meta-analysis | Journal of Occupational Health Psychology | Article | 553 | 46 | 2008 | USA |
| 9 | Counterproductive work behavior (CWB) in response to job stressors and organizational justice: Some mediator and moderator tests for autonomy and emotions | Journal of Vocational Behavior | Article | 541 | 28 | 2001 | USA |
| 10 | Job stress, employee health, and organizational-effectiveness - facet analysis, model, and literature-review | Personnel Psychology | Article | 520 | 12 | 1978 | USA |
| 11 | Job strain as a risk factor for coronary heart disease: A collaborative meta-analysis of individual participant data | Lancet | Article | 511 | 64 | 2012 | England |
| 12 | Workplace stress in nursing: a literature review | Journal of Advanced Nursing | Article | 507 | 30 | 2003 | England |
| 13 | Two alternative job stress models and the risk of coronary heart disease | American Journal of Public Health | Article | 504 | 23 | 1998 | England |
| 14 | Should negative affectivity remain an unmeasured variable in the study of job stress | Journal of Applied Psychology | Article | 489 | 15 | 1988 | USA |
| 15 | The benefits of interventions for work-related stress | American Journal of Public Health | Article | 478 | 25 | 2001 | Netherlands |
| 16 | Is job strain a major source of cardiovascular disease risk? | Scandinavian Journal of Work Environment & Health | Review | 472 | 30 | 2004 | Sweden |
| 17 | A meta-analysis of work demand stressors and job performance: examining main and moderating effects | Personnel Psychology | Review | 465 | 39 | 2008 | Israel |
| 18 | Occupational stress - its causes and consequences for job-performance | Journal of Applied Psychology | Article | 458 | 13 | 1986 | USA |
| 19 | Changes in the serum cholesterol and blood clotting time in men subjected to cyclic variation of occupational stress | Circulation | Article | 451 | 7 | 1958 | USA |
| 20 | Why negative affectivity should not be controlled in job stress research: Don't throw out the baby with the bath water | Journal of Organizational Behavior | Article | 390 | 20 | 2000 | USA |
| 21 | Impact of structural and psychological empowerment on job strain in nursing work settings - expanding Kanter's model | Journal of Nursing Administration | Article | 374 | 20 | 2001 | Canada |
| 22 | The personal costs of citizenship behavior: The relationship between individual initiative and role overload, job stress, and work-family conflict | Journal of Applied Psychology | Article | 373 | 25 | 2005 | USA |
| 23 | Job stress, incivility, and counterproductive work behavior (CWB): the moderating role of negative affectivity | Journal of Organizational Behavior | Article | 368 | 25 | 2005 | USA |
| 24 | Combined effects of job strain and social-isolation on cardiovascular-disease morbidity and mortality in a random sample of the Swedish male working population | Scandinavian Journal of Work Environment & Health | Article | 367 | 12 | 1989 | USA |
| 25 | Job strain, effort-reward imbalance and employee wellbeing: A large-scale cross-sectional study | Social Science & Medicine | Article | 359 | 18 | 2000 | Netherlands |
| 26 | The relationship between job strain, workplace diastolic blood-pressure, and left-ventricular mass index - results of a case-control study | JAMA-Journal of the American Medical Association | Article | 344 | 11 | 1990 | USA |
| 27 | Psychosocial work environment and stress-related disorders, a systematic review | Occupational Medicine-Oxford | Article | 339 | 34 | 2010 | Netherlands |
| 28 | Cross-functional project groups in research and new product development: diversity, communications, job stress, and outcomes | Academy of Management Journal | Article | 338 | 18 | 2001 | USA |
| 29 | Perceived teacher self-efficacy as a predictor of job stress and burnout: mediation analyses | Applied Psychology-An International Review-Psychologie Appliquee-Revue Internationale | Article | 335 | 28 | 2008 | Germany |
| 30 | Job stressors, personality and burnout in primary school teachers | British Journal of Educational Psychology | Article | 334 | 26 | 2007 | Greece |
| 31 | Job strain, job demands, decision latitude, and risk of coronary heart disease within the Whitehall II study | Journal of Epidemiology and Community Health | Article | 330 | 19 | 2003 | England |
| 32 | Relation of job stressors to affective, health, and performance outcomes - a comparison of multiple data sources | Journal of Applied Psychology | Article | 329 | 10 | 1988 | USA |
| 33 | Acute psychological stress reduces working memory-related activity in the dorsolateral prefrontal cortex | Biological Psychiatry | Article | 324 | 29 | 2009 | Netherlands |
| 34 | Efficacy beliefs as a moderator of the impact of work-related stressors: a multilevel study | Journal of Applied Psychology | Article | 321 | 15 | 1999 | USA |
| 35 | Work-related stress and depressive disorders | Journal of Psychosomatic Research | Article | 311 | 16 | 2001 | Australia |
| 36 | Changes in job strain in relation to changes in physiological-state - A longitudinal-study | Scandinavian Journal of Work Environment & Health | Article | 310 | 10 | 1988 | Sweden |
| 37 | The effects of perceived co-worker involvement and supervisor support on service provider role stress, performance and job satisfaction | Journal of Retailing | Article | 304 | 13 | 1996 | USA |
| 38 | Mental-health, job-satisfaction, and job stress among general-practitioners | British Medical Journal | Article | 303 | 10 | 1989 | England |
| 39 | A systematic review of the job-stress intervention evaluation literature, 1990-2005 | International Journal of Occupational and Environmental Health | Article | 295 | 23 | 2007 | Australia |
| 40 | Recovery from job stress: The stressor-detachment model as an integrative framework | Journal of Organizational Behavior | Review | 290 | 58 | 2015 | Germany |
| 41 | Occupational stress among health-care workers - A test of the job demands-control model | Journal of Organizational Behavior | Article | 285 | 9 | 1988 | USA |
| 42 | The demands-control model of job strain: A more specific test | Journal of Occupational and Organizational Psychology | Article | 274 | 11 | 1996 | England |
| 43 | Organizational determinants of job stress | Organizational Behavior and Human Performance | Article | 270 | 7 | 1983 | USA |
| 44 | Effects of stressful job demands and control on physiological and attitudinal outcomes in a hospital setting | Academy of Management Journal | Article | 268 | 10 | 1993 | USA |
| 45 | Job-related stress, social support, and burnout among classroom teachers | Journal of Applied Psychology | Article | 263 | 8 | 1987 | USA |
| 46 | Coping with job stress - measures and future-directions for scale development | Journal of Applied Psychology | Article | 261 | 8 | 1986 | USA |
| 47 | Exposure to job stress - a new psychometric instrument | Scandinavian Journal of Work Environment & Health | Article | 260 | 8 | 1988 | USA |
| 48 | Participation in decision-making as a strategy for reducing job-related strain | Journal of Applied Psychology | Article | 256 | 7 | 1983 | USA |
| 49 | The consequences of emotional labor: Effects on work stress, job satisfaction, and well-being | Motivation and Emotion | Article | 253 | 12 | 1999 | USA |
| 50 | Relief from job stressors and burnout: Reserve service as a respite | Journal of Applied Psychology | Article | 253 | 12 | 1998 | Israel |
| 51 | Can work make you sick? A meta-analysis of the relationships between job stressors and physical symptoms | Work and Stress | Article | 252 | 28 | 2011 | USA |
| 52 | The contribution of personality-traits, negative affectivity, locus of control and type-A to the subsequent reports of job stressors and job strains | Journal of Occupational and Organizational Psychology | Article | 246 | 9 | 1994 | USA |
| 53 | Association between ambulatory blood-pressure and alternative formulations of job strain | Scandinavian Journal of Work Environment & Health | Article | 243 | 9 | 1994 | USA |
| 54 | Striking a balance in boundary-spanning positions: An investigation of some unconventional influences of role stressors and job characteristics on job outcomes of salespeople | Journal of Marketing | Article | 240 | 11 | 1998 | USA |
| 55 | Aftereffects of job-related stress - families as victims | Journal of Occupational Behaviour | Article | 239 | 6 | 1982 | USA |
| 56 | Burnout and turnover intention among social workers: Effects of role stress, job autonomy and social support | Administration in Social Work | Article | 238 | 20 | 2008 | USA |
| 57 | Working in a majority context: A structural model of heterosexism as minority stress in the workplace | Journal of Counseling Psychology | Article | 236 | 11 | 1999 | USA |
| 58 | Leadership styles, mentoring functions received, and job-related stress: A conceptual model and preliminary study | Journal of Organizational Behavior | Review | 233 | 12 | 2000 | USA |
| 59 | Work stressors and coworker support as predictors of individual strain and job performance | Journal of Organizational Behavior | Article | 231 | 12 | 2000 | USA |
| 60 | Stress on and off the job as related to sex and occupational-status in white-collar workers | Journal of Organizational Behavior | Article | 231 | 7 | 1989 | Sweden |
| 61 | Occupational stress in universities: Staff perceptions of the causes, consequences and moderators of stress | Work and Stress | Article | 230 | 12 | 2001 | Australia |
| 62 | Decision latitude, job strain, and myocardial infarction: A study of working men in Stockholm | American Journal of Public Health | Article | 230 | 10 | 1998 | Sweden |
| 63 | A review of empirical studies on the model of effort-reward imbalance at work: Reducing occupational stress by implementing a new theory | Social Science & Medicine | Review | 229 | 14 | 2004 | Japan |
| 64 | Prospective effect of job strain on general and central obesity in the Whitehall II study | American Journal of Epidemiology | Article | 227 | 17 | 2007 | England |
| 65 | Minimizing strain and maximizing learning: The role of job demands, job control, and proactive personality | Journal of Applied Psychology | Article | 227 | 11 | 1999 | Australia |
| 66 | Job scope and stress - can job scope be too high | Academy of Management Journal | Article | 227 | 9 | 1995 | Canada |
| 67 | School climate and social-emotional learning: Predicting teacher stress, job satisfaction, and teaching efficacy | Journal of Educational Psychology | Article | 226 | 28 | 2012 | Canada |
| 68 | Effective and viable mind-body stress reduction in the workplace: A randomized controlled trial | Journal of Occupational Health Psychology | Article | 222 | 28 | 2012 | USA |
| 69 | Cancer care workers in Ontario: prevalence of burnout, job stress and job satisfaction | Canadian Medical Association Journal | Article | 222 | 11 | 2000 | Canada |
| 70 | Job strain and anger expression predict early morning elevations in salivary cortisol | Psychosomatic Medicine | Article | 221 | 11 | 2000 | England |
| 71 | A longitudinal study of job strain and ambulatory blood pressure: results from a three-year follow-up | Psychosomatic Medicine | Article | 216 | 10 | 1998 | USA |
| 72 | Occupational stress and health among factory-workers | Journal of Health and Social Behavior | Article | 216 | 5 | 1979 | USA |
| 73 | The relationship between job stress, burnout and clinical depression | Journal of Affective Disorders | Article | 215 | 13 | 2003 | Greece |
| 74 | Emotion work and job stressors and their effects on burnout | Psychology & Health | Article | 215 | 11 | 2001 | Germany |
| 75 | Job stressors, emotional exhaustion, and need for recovery: a multi-source study on the benefits of psychological detachment | Journal of Vocational Behavior | Article | 214 | 21 | 2010 | Germany |
| 76 | Divergent effects of job control on coping with work stressors: the key role of self-efficacy | Academy of Management Journal | Article | 211 | 9 | 1997 | USA |
| 77 | Occupational stress, burnout, and health in teachers: a methodological and theoretical analysis | Review of Educational Research | Review | 209 | 10 | 1998 | USA |
| 78 | The association of job strain and health behaviours in men and women | International Journal of Epidemiology | Article | 208 | 9 | 1997 | USA |
| 79 | Relation between job strain, alcohol, and ambulatory blood-pressure | Hypertension | Article | 206 | 7 | 1992 | USA |
| 80 | Stress and psychiatric disorder in healthcare professionals and hospital staff | Lancet | Article | 205 | 10 | 2000 | England |
| 81 | Relations between stress and work outcomes: the role of felt challenge, job control, and psychological strain | Journal of Vocational Behavior | Article | 204 | 13 | 2004 | USA |
| 82 | Job stress, job performance, and social support among hospital nurses | Journal of Nursing Scholarship | Article | 200 | 13 | 2004 | Jordan |
| 83 | Windows in the workplace - sunlight, view, and occupational stress | Environment and Behavior | Article | 200 | 9 | 1998 | England |
| 84 | Shift work and metabolic syndrome: respective impacts of job strain, physical activity, and dietary rhythms | Chronobiology International | Article | 199 | 18 | 2009 | France |
| 85 | Conflicts in the work-family interface: links to job stress, customer service employee performance, and customer purchase intent | Journal of Marketing | Article | 199 | 13 | 2005 | USA |
| 86 | Management standards' work-related stress in the UK: practical development | Work and Stress | Article | 198 | 12 | 2004 | England |
| 87 | Occupational stress and coronary heart disease - review and theoretical integration | Journal of Health and Social Behavior | Article | 198 | 4 | 1974 | USA |
| 88 | Work stress, weight gain and weight loss: Evidence for bidirectional effects of job strain on body mass index in the Whitehall II study | International Journal of Obesity | Article | 197 | 14 | 2006 | Finland |
| 89 | Neutralizing job stressors: Political skill as an antidote to the dysfunctional consequences of role conflict | Academy of Management Journal | Article | 197 | 12 | 2004 | USA |
| 90 | Occupational stress and variation in cigarette, coffee, and alcohol-consumption | Journal of Health and Social Behavior | Article | 196 | 5 | 1981 | USA |
| 91 | Relative significance of heredity, diet and occupational stress in coronary heart disease of young adults - based on an analysis of 100 patients between the ages of 25 and 40 years and a similar group of 100 normal control subjects | American Journal of the Medical Sciences | Article | 196 | 3 | 1958 | USA |
| 92 | An investigation of health complaints and job stress in video display operations | Human Factors | Article | 193 | 5 | 1981 | USA |
| 93 | Job stress and job-performance controversy - an empirical-assessment | Organizational Behavior and Human Performance | Article | 189 | 5 | 1984 | Canada |
| 94 | Job stress and burnout among correctional officers: a literature review | International Journal of Stress Management | Article | 188 | 9 | 2000 | Netherlands |
| 95 | Coping with job stress and burnout in the human-services | Journal of Personality and Social Psychology | Article | 185 | 5 | 1984 | USA |
| 96 | Coping with job stress - A conceptual evaluation framework for coping measures | Journal of Organizational Behavior | Article | 180 | 6 | 1992 | USA |
| 97 | Assessment of job stress dimensions based on the job demands-control model of employees of telecommunication and electric power companies in Japan: Reliability and validity of the Japanese version of the job content questionnaire | International Journal of Behavioral Medicine | Article | 177 | 7 | 1995 | Japan |
| 98 | Psychological stress in the workplace | Journal of Social Behavior and Personality | Article | 177 | 6 | 1991 | USA |
| 99 | Extending the challenge-hindrance model of occupational stress: The role of appraisal | Journal of Vocational Behavior | Article | 174 | 19 | 2011 | USA |
| 100 | learning how to recover from job stress: effects of a recovery training program on recovery, recovery-related self-efficacy, and well-being | Journal of Occupational Health Psychology | Article | 174 | 19 | 2011 | Germany |
